# Preparation, Characterisation, and Fresh-Keeping Application of Pullulan/Konjac Glucomannan/Low-Acyl Gellan Gum Ternary Edible Composite Films for Extending the Shelf Life of Plums

**DOI:** 10.3390/foods15183235

**Published:** 2026-09-13

**Authors:** Peng Huang, Lei Cao, Mingzhen Li, Fang Wu, Yujia Xia, Shuyi Li, Tongtong Ye, Shuxin Peng, Lin Ye, Pinyao Zhao, Wen Qin

**Affiliations:** 1 Department of Quality Management and Inspection, Yibin University, 8 Jiusheng Road, Yibin 644000, China; 15283846855@163.com (L.C.); 18782218105@163.com (Y.X.); 19383029801@163.com (S.L.); 230404154@stu.yibinu.edu.cn (S.P.); yelinlyn1992@gmail.com (L.Y.); zhaopy@yibinu.edu.cn (P.Z.); 2 Engineering Research Centre for Standardization, Inspection and Testing of Agricultural and Food Products, Sichuan Higher Education Institutions, Yibin University, Yibin 644000, China; 18080988236@163.com (F.W.); titieee@163.com (T.Y.); 3 College of Materials Engineering, Fujian Agriculture and Forestry University, No. 63 Xiyuangong Road, Minhou County, Fuzhou 350002, China; 4 College of Food Science, Sichuan Agricultural University, 46 Xinkang Road, Ya’an 625014, China

**Keywords:** pullulan, konjac glucomannan, low-acyl gellan gum, edible composite films, response surface methodology, plums, preservation

## Abstract

The escalating environmental burden of conventional plastic food packaging has intensified the search for sustainable, biodegradable alternatives. However, single-component polysaccharide edible films often exhibit suboptimal mechanical and barrier properties, which limits their practical applicability. In this study, pullulan (PUL), konjac glucomannan (KGM) and low-acyl gellan gum (LGG) were blended as film-forming substrates with glycerol as a plasticiser. Single-factor experiments combined with Box–Behnken response surface methodology (RSM) were employed to optimise the formulation of ternary edible composite films. The effects of PUL, KGM and LGG concentrations on mechanical properties and water vapour permeability (WVP) were systematically investigated. The optimised film was comprehensively characterised for microstructure, physical barrier performance, antioxidant activity, soil disintegration, bacteriostatic capacity and plum preservation efficiency. The optimal formulation comprised 1.43 g/100 mL PUL, 0.24 g/100 mL KGM and 0.48 g/100 mL LGG. Under these conditions, the composite film exhibited a transparent and smooth appearance, with a continuous and dense internal microstructure. The comprehensive performance score (K) reached 0.805 ± 0.013, with a WVP of 2.75 ± 0.17 g·μm/(m^2^·h·kPa), light transmittance of 89.93% ± 1.26%, tensile strength (TS) of 3.05 ± 0.14 N/cm^2^, elongation at break (EB) of 64.57% ± 0.58% and DPPH radical scavenging activity of 34.12% ± 0.96%. Storage trials on two plum varieties demonstrated that the composite coating effectively mitigated fruit weight loss, retarded peel browning and pulp softening, and maintained superior sensory quality throughout storage. Overall, the PUL/KGM/LGG ternary edible composite film exhibited favourable mechanical strength, promising water vapour barrier properties and moderate antioxidant activity, suggesting its potential as a useful reference for the further development and industrial adoption of biodegradable edible films in fruit and vegetable preservation.

## 1. Introduction

The escalating global plastic pollution crisis has intensified the search for sustainable alternatives to petroleum-based packaging materials [1]. Edible films and coatings derived from natural biopolymers have emerged as promising candidates due to their soil disintegration, biocompatibility and ability to extend the shelf life of perishable foods [2,3]. Among the various biopolymers explored, polysaccharides are particularly attractive owing to their abundance, favourable film-forming properties and good gas barrier characteristics [4,5].

Pullulan (PUL), a neutral linear polysaccharide produced by *Aureobasidium pullulans*, possesses outstanding film-forming ability, high transparency and excellent oxygen barrier properties [6,7]. However, PUL films are inherently hydrophilic and exhibit limited mechanical strength, which restricts their practical application [8]. Konjac glucomannan (KGM), a water-soluble polysaccharide extracted from the tuber of Amorphophallus konjac, has been widely investigated for edible film development due to its excellent film-forming capacity, soil disintegration and biocompatibility [9,10]. Nevertheless, pure KGM films suffer from high water vapour permeability and poor mechanical properties, primarily owing to their strong hydrophilicity [11]. Low-acyl gellan gum (LGG), a microbial exopolysaccharide produced by Sphingomonas elodea, forms strong, brittle gels with considerable mechanical strength and thermal stability [12,13].

Previous studies have demonstrated the benefits of blending polysaccharides to enhance film performance. Yan et al. [14] reported that KGM/PUL blend films exhibited improved mechanical and barrier properties attributable to hydrogen bonding interactions between the two polymers, with optimal performance achieved at a KGM:PUL ratio of 2:1. Similarly, Zhou et al. [15] investigated the effect of hydroxypropyl-β-cyclodextrin and lecithin co-stabilised nanoemulsions on KGM/PUL films and found that the incorporation of nanoemulsions improved structural integrity and controlled release properties. These studies confirm that binary blending can yield measurable enhancements in film performance.

Beyond binary systems, ternary composite formulations have demonstrated even greater potential. Systems, such as sodium alginate/low-methoxyl pectin/locust bean gum films and gellan gum/chitosan/melatonin films, have shown superior integrated performance compared to binary or single-component systems. Gellan gum-based films have been extensively studied for their excellent film-forming properties and biocompatibility [12,13], with recent reviews highlighting their great application potential in food preservation. Notably, binary blends such as KGM/pullulan [14] and pullulan/gellan gum combinations have demonstrated significant improvements in mechanical properties, barrier performance and preservation efficacy relative to single-component films. Nevertheless, binary systems often face an inherent trade-off between rigidity and flexibility: enhancing tensile strength through the incorporation of rigid polymers typically compromises extensibility, while prioritising flexibility tends to increase water vapour permeability [12,14]. Consequently, we hypothesised that the introduction of a third complementary polysaccharide could overcome this limitation by forming a synergistic interpenetrating network. Huang et al. [16] developed KGM/LGG edible coatings containing thymol microcapsules and demonstrated that such coatings effectively inhibited postharvest softening and prolonged the shelf life of blueberries by regulating cell wall polysaccharide disassembly. More recently, Yao et al. [17] developed pullulan/gellan gum biofilms loaded with astaxanthin nanoemulsion and demonstrated that such films effectively extended strawberry shelf life to 18 days, confirming the compatibility of pullulan and gellan gum as a film-forming matrix. Collectively, these findings highlight the potential of polysaccharide-based systems for fruit preservation; however, the systematic investigation of a ternary PUL/KGM/LGG system—particularly its comparative advantages over binary counterparts—remains unexplored.

Based on the complementary structural features of the three polysaccharides—PUL providing chain flexibility and film-forming ability, KGM contributing chain entanglement and hydrophilicity modulation via its branched β-(1→4)-linked backbone, and LGG offering network rigidity through double-helix junctions formed upon cation-mediated aggregation—we hypothesised that a ternary PUL/KGM/LGG system might form a synergistic interpenetrating polymer network through extensive hydrogen bonding between hydroxyl and carboxyl groups, potentially yielding superior mechanical strength, barrier properties and antioxidant activity compared to binary or single-component systems. Specifically, we anticipated that the flexible PUL chains would act as molecular connectors between the rigid KGM and LGG domains, thereby preventing macroscopic phase separation and facilitating stress distribution during deformation. Furthermore, we hypothesised that this ternary matrix could address the key preservation challenges of stone fruits such as plums—namely moisture loss leading to shrivelling, oxidative browning mediated by polyphenol oxidase, and softening resulting from cell wall disintegration—via a combination of moisture barrier, oxygen barrier and inherent antioxidant functions. To test these hypotheses, we employed Box–Behnken response surface methodology to systematically optimise the ternary formulation and comprehensively characterised the resulting film’s structure–function relationships. To the best of our knowledge, no systematic investigation of PUL/KGM/LGG ternary composite films-particularly those employing response surface methodology (RSM) for optimisation-has been previously reported. Furthermore, the application of such ternary polysaccharide composite films specifically for plum preservation remains underexplored. The present study therefore addresses these gaps by: (1) optimising the PUL/KGM/LGG ternary formulation using single-factor experiments and Box–Behnken RSM; (2) comprehensively characterising the optimised film’s microstructure, mechanical properties, barrier performance, antioxidant activity, and soil disintegration; and (3) evaluating its preservation efficacy on two plum cultivars under ambient storage conditions.

RSM is a statistical technique widely used to optimise multiple variables and evaluate interactive effects in food packaging research [18]. The Box–Behnken design, a type of RSM, offers advantages in terms of efficiency and reduced experimental runs compared to central composite designs [19]. This approach has been successfully applied to optimise polysaccharide-based edible films, including sodium alginate/graphene nanoplatelet nanocomposite films and corn starch films [20]. RSM has also been employed to optimise other polysaccharide-based edible film formulations, further supporting the versatility of this approach [21].

Plums (Prunus salicina) are highly perishable fruits prone to rapid quality deterioration during postharvest storage, including weight loss, softening, browning and microbial decay [22]. Edible coatings have shown potential in extending plum shelf life by regulating respiration and moisture loss [23]. Recent studies have explored various polysaccharide-based coatings for plum preservation, including buckwheat starch with xanthan gum and lemongrass essential oil [24], as well as hydroxypropyl methylcellulose-based coatings. Multifunctional nanocomposite films based on carboxymethyl cellulose have also been shown to extend the shelf life of plums by up to 25 days. Nevertheless, the application of ternary polysaccharide composite films for plum preservation remains underexplored.

Therefore, this study aimed to optimise the PUL/KGM/LGG ternary edible composite film formulation using single-factor experiments and Box–Behnken response surface methodology, to comprehensively characterise the optimised film in terms of microstructure, mechanical properties, barrier properties and antioxidant activity, and to evaluate its preservation efficacy on plums during ambient storage. Through this systematic approach, we sought to validate the proposed synergistic mechanism and provide a practical reference for the development of biodegradable edible films for fruit preservation.

## 2. Materials and Methods

### 2.1. Materials and Reagents

Food-grade PUL was obtained from Shandong Mimei Biotechnology Co., Ltd. (Weifang, China). Food-grade KGM was procured from Hubei Qiangsen Konjac Technology Co., Ltd. (Ezhou, China), and food-grade LGG was supplied by Xinjiang Fuyang Biotechnology Co., Ltd. (Urumqi, China). Glycerol (food grade) was obtained from Fengyi Oil Technology Co., Ltd. (Tianjin, China). 1,1-Diphenyl-2-picrylhydrazyl (DPPH, analytical grade) was sourced from Fuzhou Feijing Biotechnology Co., Ltd. (Fuzhou, China). All other reagents were of analytical grade and purchased from Shanghai Aladdin Biochemical Technology Co., Ltd. (Shanghai, China).

Commercially mature plums (cultivars: ‘Yinhongli’ and ‘Qingcuili’) at 80% maturity were harvested from commercial orchards in Yibin, Sichuan Province, China. To ensure homogeneity, fruits with uniform colour and size, and free from physical defects or pest infestation, were selected for preservation experiments.

### 2.2. Composite Film Preparation

The composite film was prepared following the method described by Huang et al. [16], with slight modifications. Briefly, appropriate amounts of PUL, KGM, and LGG were accurately weighed, followed by the addition of glycerol at a concentration of 1% (*w*/*v*) as a plasticiser, based on preliminary experiments and previous literature [14,15]. The mixture was placed in a water bath maintained at 60 °C and continuously stirred at 1200 rpm for 30 min using a mechanical stirrer (Model 79-1, Changzhou Henglong Instrument Co., Ltd., Changzhou, China). The mixture was then subjected to ultrasonic treatment at 25 kHz for 30 min to remove bubbles in a dual-frequency ultrasonic cleaner (Model SB-4200DTS, Ningbo Scientz Biotechnology Co., Ltd., Ningbo, China). A measured volume (20 mL) of the film-forming solution was slowly poured onto preheated Petri dishes (90 mm diameter) and spread evenly, then dried in an electric blast drying oven (Model 202-OA, Shanghai Shangpu Instrument Equipment Co., Ltd., Shanghai, China) at 60 °C for 5 h. The resulting dried films were conditioned at 25 °C and 50% relative humidity for at least 24 h prior to characterisation.

### 2.3. Single-Factor Experimental Design

The optimal concentrations of PUL, KGM, and LGG were determined using a single-factor experimental design. The factor ranges for each component were selected based on preliminary experiments and literature precedents. Specifically, the PUL concentration range (0–1.9 g/100 mL) was chosen to encompass the concentrations reported in PUL-based edible film studies [14], while ensuring solution viscosity remained manageable for film casting. The KGM range (0–0.35 g/100 mL) and LGG range (0–0.7 g/100 mL) were selected based on previous KGM/LGG composite film formulations [16] and preliminary trials identifying concentrations that produced handleable films with measurable properties. The base formulation of the film-forming solution was established as PUL 1.5%, KGM 0.25%, and LGG 0.5%. This baseline was selected based on preliminary experiments as the central point that produced a handleable film with balanced properties. Tensile strength (TS), elongation at break (EB), and water vapour permeability (WVP) were selected as indicators to evaluate the overall performance through membership degree analysis. The influence of different concentrations of each component-PUL (0%, 1.1%, 1.3%, 1.5%, 1.7%, and 1.9%), KGM (0%, 0.15%, 0.2%, 0.25%, 0.3%, and 0.35%), and LGG (0%, 0.3%, 0.4%, 0.5%, 0.6%, and 0.7%)-on the properties of the composite films was investigated. All composite films were prepared following the procedure outlined in Section 2.2.

### 2.4. Box-Behnken Response Surface Design

**Figure 1:**
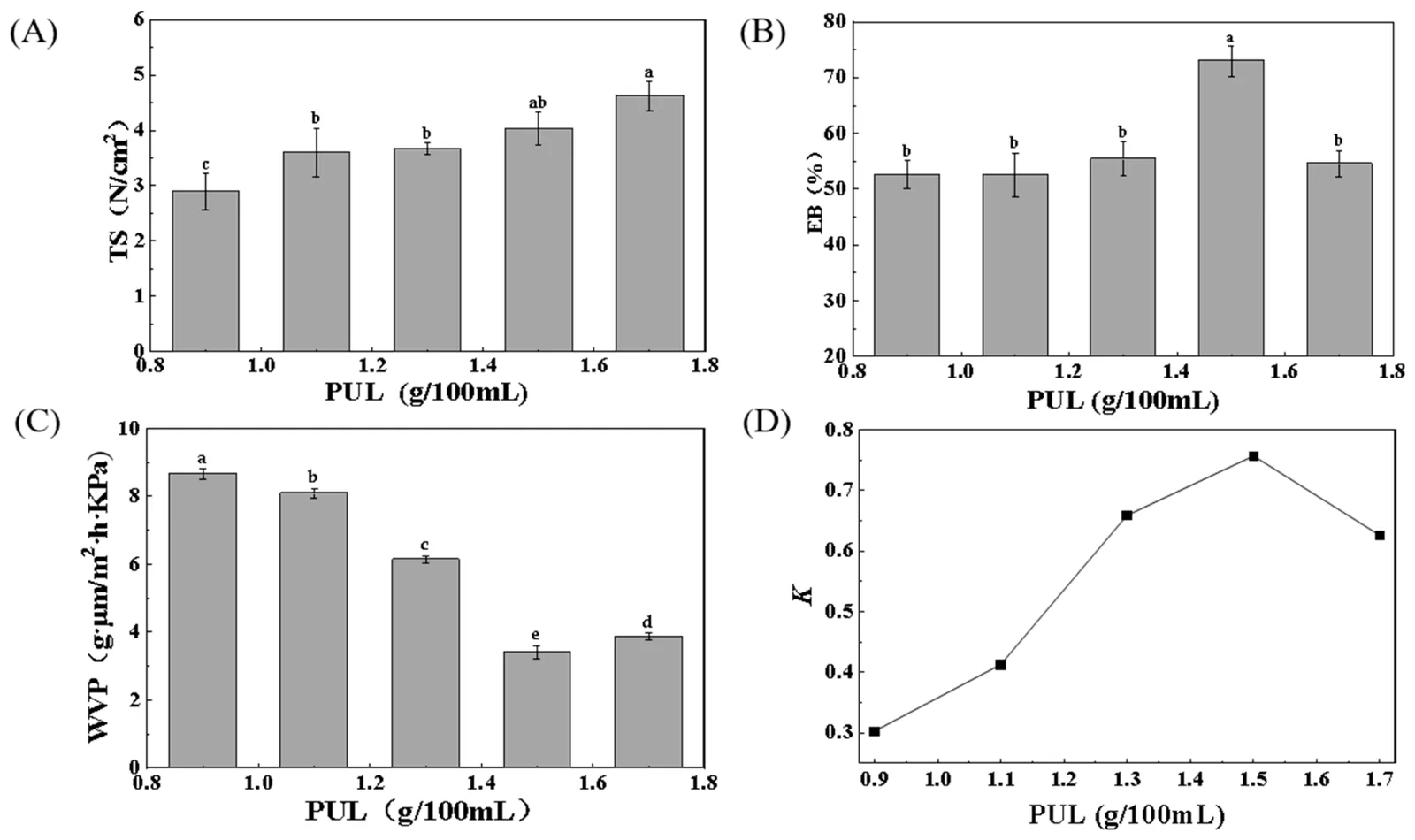
Effects of PUL addition level on tensile strength (**A**), elongation at break (**B**), water vapour permeability coefficient (**C**) and comprehensive performance (**D**) of composite films. Different letters indicate significant differences among concentration treatments (*p* < 0.05).

**Figure 2:**
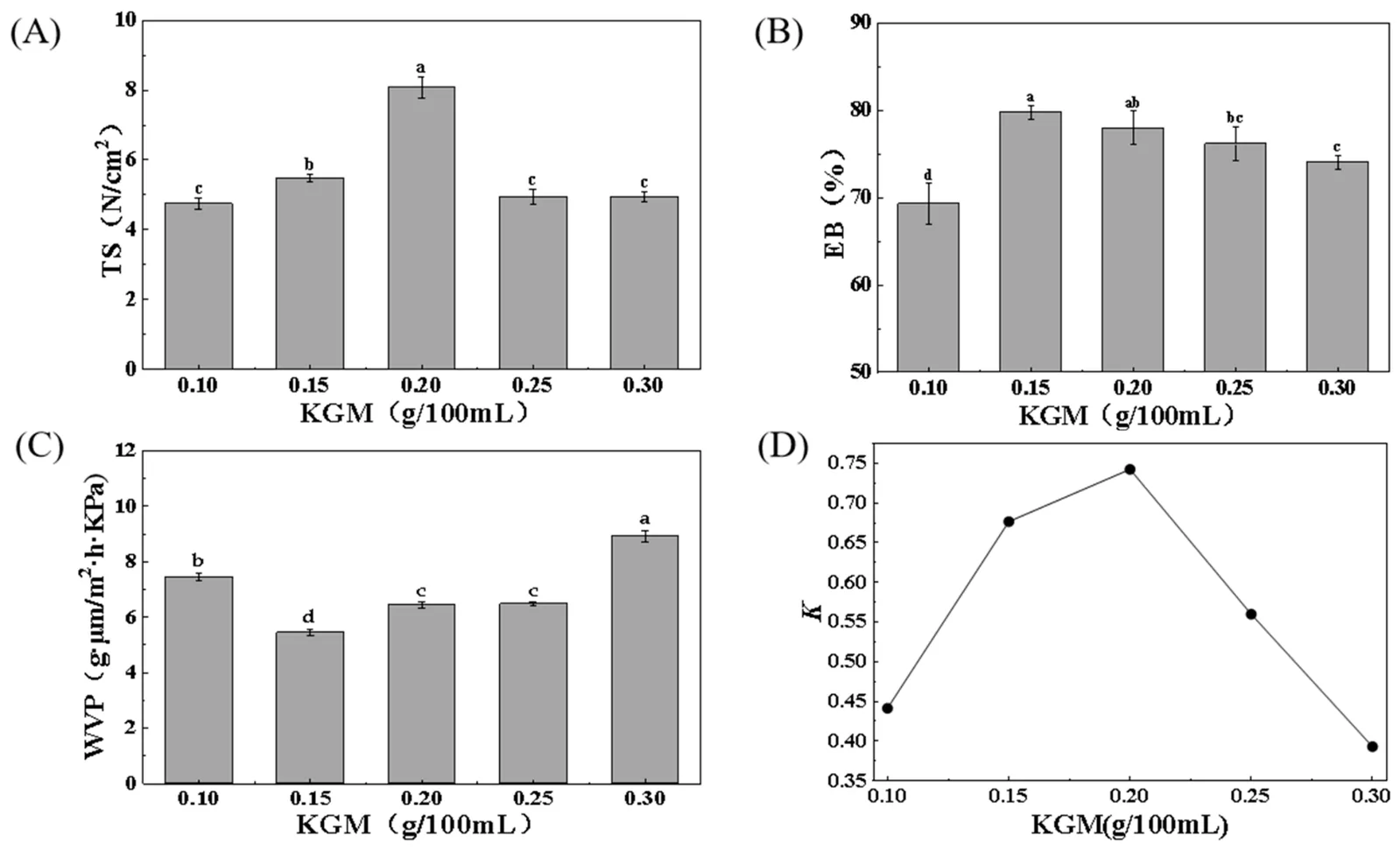
Effects of KGM addition level on tensile strength (**A**), elongation at break (**B**), water vapour permeability coefficient (**C**) and comprehensive performance (**D**) of composite films. Different letters indicate significant differences among concentration treatments (*p* < 0.05).

**Figure 3:**
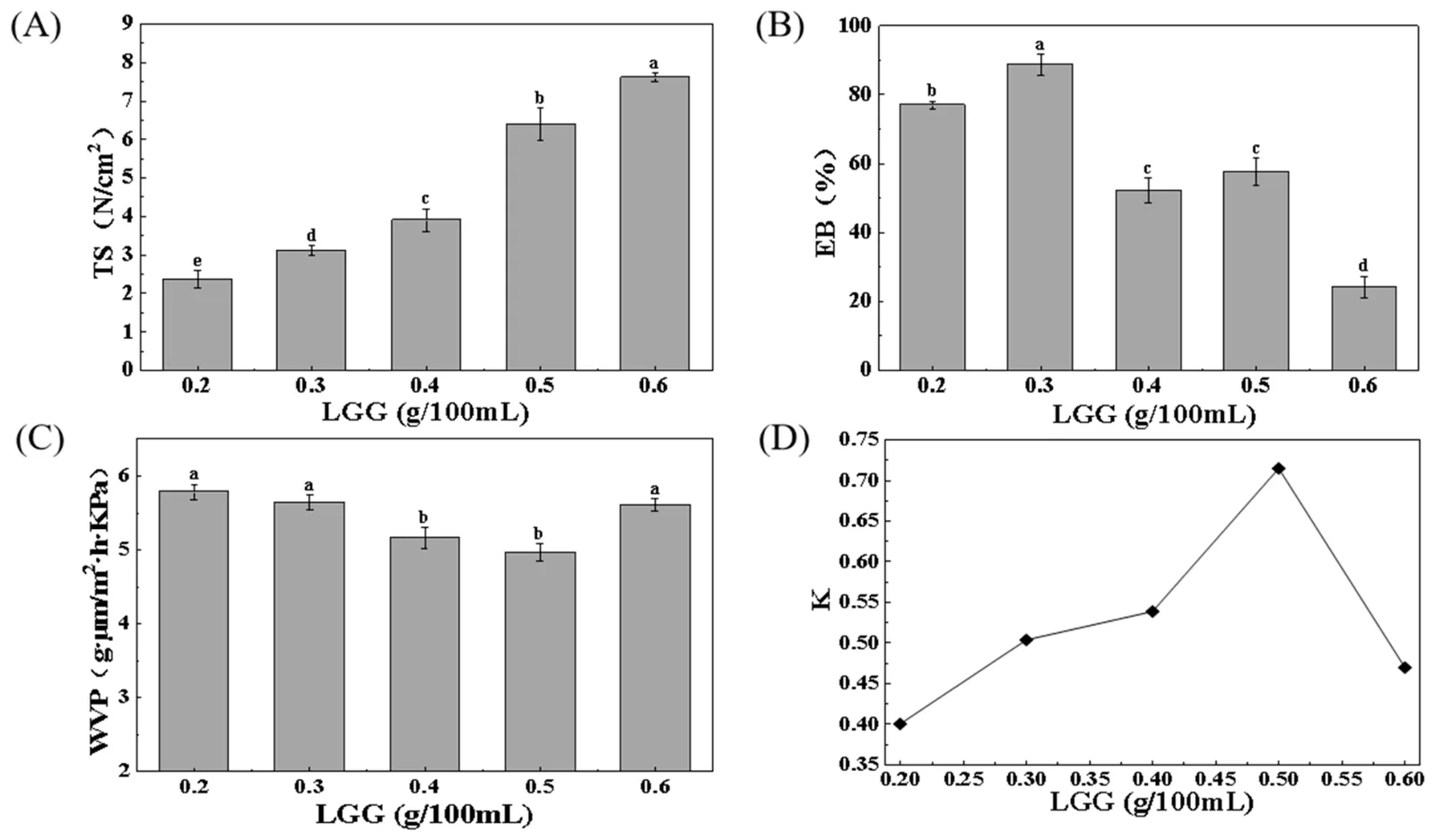
Effects of LGG addition level on tensile strength (**A**), elongation at break (**B**), water vapour permeability coefficient (**C**) and comprehensive performance (**D**) of composite films. Different letters indicate significant differences among concentration treatments (*p* < 0.05).

Based on the comprehensive evaluation results of single-factor experiments, the appropriate addition amounts of each raw material were screened out using response surface methodology (RSM), which has been widely employed for optimising edible film formulations [25]. With the aid of Design-Expert software (Version 13, Stat-Ease, Inc., Minneapolis, MN, USA), a three-factor and three-level response surface methodology model was established via Box–Behnken design. The factor levels for the Box–Behnken design were determined based on the single-factor experimental results: the concentrations that gave the highest comprehensive K-values for each component were used as centre points, with adjacent concentrations chosen as the low and high levels. Specifically, as shown in Figures 1–3 and discussed in Section 3.1, the optimal concentrations identified from single-factor experiments were PUL 1.5 g/100 mL, KGM 0.20 g/100 mL, and LGG 0.5 g/100 mL. These values served as the centre points (level 0) for the Box–Behnken design. The adjacent concentrations (PUL 1.3 and 1.7 g/100 mL; KGM 0.15 and 0.25 g/100 mL; LGG 0.4 and 0.6 g/100 mL) were selected as the low (−1) and high (+1) levels, respectively, to capture the curvature of the response surface around the optimal regions. The independent variables were PUL concentration (A: 1.3, 1.5, 1.7 g/100 mL), KGM concentration (B: 0.15, 0.20, 0.25 g/100 mL), and LGG concentration (C: 0.4, 0.5, 0.6 g/100 mL). The comprehensive performance K-value, calculated using fuzzy comprehensive evaluation (Section 2.8), served as the response variable. The experimental design comprised 15 runs with three centre points.

### 2.5. Characterisation of Films

Composite films with varying polysaccharide combinations (LGG, KGM/LGG, PUL/LGG, and PUL/KGM/LGG) were prepared according to the method described in Section 2.2, and their compositions are detailed in Table A1.

#### 2.5.1. Films Thickness

Film thickness was measured following the procedure of Kumar et al. [26], with minor adjustments. Specimens were folded into eight-layer stacks, and the total thickness was recorded with a vernier caliper (0.01 mm precision). The thickness of a single layer was obtained by dividing the total thickness by the number of layers. All measurements were conducted in triplicate, and the mean was taken as the representative value.

#### 2.5.2. Mechanical Properties

TS and EB were evaluated using a texture analyser (TA-XTC-18, Baosheng Co., Shanghai, China) following the procedure of Shah et al. [27] with minor adjustments. Film specimens (40 × 10 mm) were tested at a crosshead speed of 1 mm/s with an initial grip separation of 30 mm. TS (MPa) and EB (%) were computed as:

$$TS=\frac{F}{W\times x}$$

$$EB(\%)=\frac{L}{L_{0}}\times 100$$

where *F* is the maximum force (N), *W* is the specimen width (mm), *x* is the film thickness (mm), *L* is the elongation at break (mm), and *L*_0_ is the initial gauge length (mm). Triplicate measurements were performed for each sample.

#### 2.5.3. WVP

Water Vapour Permeability (WVP) was determined using the desiccant method according to Saini et al. [28] with modifications. Film specimens were sealed onto glass vials containing 15 mL distilled water and placed in a desiccator with silica gel at 25 °C. Weight loss was recorded every 4 h for 12 h. WVP was calculated as:

$$WVP=\frac{\Delta w\times x}{t\times A\times \Delta p}$$

where Δ*w* is the weight change (g), *x* is the film thickness (μm), *t* is the time interval (h), *A* is the permeation area (m^2^), and Δ*p* is the water vapour pressure difference (2.915 kPa at 25 °C).

#### 2.5.4. Light Transmittances

Film transparency was evaluated by measuring light transmittance at 600 nm using a UV-visible spectrophotometer (UV-1800, Shanghai Meixi Instruments Co., Ltd., Shanghai, China) according to the method described by Huang et al. [16]. Film specimens (10 mm × 40 mm) were placed against the cuvette wall, and an empty cuvette served as the blank.

#### 2.5.5. Scanning Electron Microscopy

Surface morphology of films was examined using a scanning electron microscope (SEM, Hitachi S-3400N, Hitachi Limited, Tokyo, Japan) at an accelerating voltage of 15 kV and 1500× magnification following the procedure described in previous studies [14]. Film specimens were sputter-coated with gold prior to observation.

#### 2.5.6. Fourier Transform Infrared Spectroscopy

Fourier Transform Infrared Spectroscopy (FTIR) spectra were recorded using a Nicolet iS50 spectrometer (Thermo Fisher Scientific, Waltham, MA, USA) in transmission mode. Film samples were ground with KBr powder (1:100, *w*/*w*) and pressed into pellets. Spectra were collected over the range of 4000–400 cm^−1^ with a resolution of 4 cm^−1^ and 32 scans.

#### 2.5.7. Bacteriostatic Activity

Bacteriostatic activity against *Escherichia coli* (*E. coli*) and *Staphylococcus aureus* (*S. aureus*) was evaluated using the growth curve method according to Ahmad et al. [29], with modifications. Film-forming solution (20 μL) was added to 96-well plates containing 160 μL Luria–Bertani (LB) medium and 20 μL bacterial suspension (OD_600_ = 1.0). Plates were incubated at 37 °C, and OD_600_ was measured every 2 h.

#### 2.5.8. DPPH Radical Scavenging Activity

Antioxidant activity was evaluated using the DPPH radical scavenging method according to Rahmawati et al. [30], with modifications. Film samples (3 g) were immersed in 25 mL distilled water for 24 h. The supernatant (3 mL) was mixed with an equal volume of DPPH ethanol solution (0.1 mM) and incubated in the dark for 30 min. Absorbance was measured at 517 nm. Scavenging activity was calculated as:

$$Scavenging\;activity(\%)=\frac{Ac-As}{Ac}\times 100$$

where *Ac* is the absorbance of the blank control; *As* is the absorbance of the mixture of sample supernatant and DPPH ethanol solution.

#### 2.5.9. Soil Disintegration Test

Film disintegration was assessed by soil burial method according to Olawade et al. [31] with modifications. Film specimens (4 cm × 4 cm) were buried 3 cm deep in soil. After 48 h, specimens were retrieved, cleaned, dried to constant weight, and weighed. Disintegration rate was calculated based on weight loss:

$$Disintegration\;rate(\%)=\frac{w_{0}-w_{1}}{w_{0}}\times 100$$

where $w_{0}$ is the initial dry mass and $w_{1}$ is the dry mass after 48 h disintegration.

### 2.6. Preservation Experiment

Plums of uniform size and maturity, free from visible defects, were randomly allocated to two groups: a control group (CK) and a PUL/KGM/LGG coating treatment group. For the treatment group, fruits were immersed in the optimised film-forming solution for 2 min, air-dried at ambient temperature (25 ± 2 °C, 60 ± 5% relative humidity), and then stored in polyethylene bags. The control group was subjected to the same procedure using distilled water instead of the coating solution. All samples were stored at 25 ± 2 °C, and quality attributes were evaluated at 2-day intervals throughout the 12-day storage period following the methodology described by Valero et al. [22].

#### 2.6.1. Determination of Weight Loss Rate

Weight loss rate (WLR) was determined using the gravimetric method as described by Dai et al. [32] with slight modifications. In each group, 10 fruits were randomly selected, tagged and weighed at the beginning of the experiment using an analytical balance (accuracy: 0.01 g). The mass was recorded every 2 days, and the weight loss rate was calculated as the percentage of mass loss relative to the initial weight.

#### 2.6.2. Sensory Quality Evaluation

Sensory quality was assessed via visual observation and photographic documentation. A panel of ten trained evaluators independently scored each fruit using a 10-point hedonic scale for the following established methodology [16,33]. Panelists assessed appearance, odor, and texture using a 10-point descriptive scale. Detailed scoring criteria are provided in Table A2. Digital images of both the external surface and the longitudinal cross-section of the fruits were captured under consistent lighting conditions at each sampling interval (every 2 days) to monitor changes in appearance, peel colour, surface shrivelling, and flesh browning.

### 2.7. Fuzzy Comprehensive Evaluation

The comprehensive performance *K*-value was calculated using fuzzy comprehensive evaluation according to Huang et al. [33] with modifications. WVP was assigned the highest weight (0.4) because water vapour barrier performance is the most critical factor affecting preservation efficacy in fruit coatings [28], as moisture loss is the primary cause of quality deterioration during ambient storage. TS and EB were assigned equal weights (0.3 each) because mechanical strength and flexibility are both essential for film handleability and integrity during application, and preliminary experiments showed that these parameters exhibited similar ranges of variation and practical importance. Three indicators-TS, EB, and WVP-were assigned weights of 0.3, 0.3, and 0.4, respectively. Membership values were calculated as:

For positive indicators (TS, EB):

$$M=\frac{X_{i}-X_{min}}{X_{max}-X_{min}}$$

For negative indicators (WVP):

$$M=1-\frac{X_{i}-X_{min}}{X_{max}-X_{min}}$$

The comprehensive *K*-value was then calculated as:

$$K={0.3M}_{TS}+{0.3M}_{EB}+{0.4M}_{MVP}$$

### 2.8. Data Analysis

All experiments were performed in triplicate, and results are expressed as mean ± standard deviation. Data were analysed using SPSS 26.0 (IBM Corp., Armonk, NY, USA) with one-way ANOVA followed by Duncan’s multiple range test (*p* < 0.05). Graphs were generated using Origin 2026 (OriginLab Corp., Northampton, MA, USA).

## 3. Results

### 3.1. Single-Factor Experiments

#### 3.1.1. Effect of PUL Concentration on Film Properties

The influence of PUL concentration on film mechanical properties and WVP is presented in Figure 1. TS increased progressively with increasing PUL concentration from 1.1% to 1.7% (*p* < 0.05), reaching a maximum at 1.7 g/100 mL (Figure 1A). This enhancement may be related to the formation of extensive hydrogen bonding networks between PUL and the other polysaccharide components, which would increase molecular chain crosslinking and film cohesion [34]. Conversely, EB exhibited a bell-shaped response, peaking at 1.5 g/100 mL (Figure 1B). The initial increase in EB may reflect improved chain mobility and intermolecular entanglements at moderate PUL concentrations, whereas the subsequent decline at higher concentrations perhaps reflects excessive chain rigidity and reduced flexibility [14].

WVP decreased substantially with increasing PUL concentration up to 1.5 g/100 mL, beyond which a slight increase was observed (Figure 1C). The reduced WVP at the optimal PUL concentration suggests the formation of a denser, more compact film structure that effectively impedes water vapour transmission [35]. The subsequent increase at higher PUL concentrations might result from structural inhomogeneities or microdefects arising from excessive polymer loading [36]. Comprehensive performance evaluation (Figure 1D) identified 1.5 g/100 mL as the optimal PUL concentration.

#### 3.1.2. Effects of KGM Concentration on Film Properties

KGM concentration exerted significant effects on all measured properties (Figure 2). Both TS and EB followed a similar bell-shaped pattern, with TS peaking at 0.20 g/100 mL and EB reaching a maximum at 0.15 g/100 mL (Figure 2A,B). The initial improvement in mechanical properties could be attributable to the abundant hydroxyl groups on KGM molecules, which may facilitate hydrogen bonding with PUL and LGG, thereby forming a more cohesive network [37]. However, excessive KGM concentrations might promote molecular aggregation and entanglement, disrupting structural homogeneity and causing stress concentration points that reduce both strength and extensibility [38].

WVP exhibited a minimum at 0.15 g/100 mL KGM (Figure 2C), which may be consistent with the formation of a dense, well-organised film structure at moderate KGM loading. The increase in WVP at higher KGM concentrations likely reflects the hydrophilic nature of KGM, which may enhance water adsorption and provide additional pathways for water vapour diffusion. The optimal KGM concentration for comprehensive performance was determined as 0.20 g/100 mL (Figure 2D).

#### 3.1.3. Effects of LGG Concentration on Film Properties

LGG addition produced distinct effects on mechanical properties (Figure 3). TS increased monotonically with LGG concentration up to 0.6 g/100 mL (Figure 3A), possibly reflecting the high intrinsic mechanical strength of LGG gels [12]. Recent studies have reported that the addition of LGG significantly increases the TS of composite films [14], confirming the reinforcing effect of LGG. In contrast, EB exhibited a maximum at 0.3 g/100 mL followed by a decline (Figure 3B). This biphasic response suggests that moderate LGG concentrations may enhance chain flexibility through uniform network formation, while higher concentrations might promote self-aggregation and microphase separation, compromising extensibility [39].

WVP decreased to a minimum at 0.5 g/100 mL LGG (Figure 3C), possibly indicating optimal barrier performance at this concentration. The subsequent increase in WVP at higher LGG concentrations could result from microstructural defects or phase separation, consistent with the observed decline in EB [40]. Comprehensive evaluation identified 0.5 g/100 mL as the optimal LGG concentration (Figure 3D). These single-factor experiments not only identified the optimal concentration of each component but also provided the basis for selecting the factor levels (central points and ranges) for the subsequent Box–Behnken response surface optimisation (Section 2.4).

### 3.2. Response Surface Methodology Experiments

#### 3.2.1. Model Fitting and Statistical Analysis

Based on the single-factor results (which identified optimal concentrations of PUL 1.5 g/100 mL, KGM 0.20 g/100 mL, and LGG 0.5 g/100 mL; Figure 1, Figure 2 and Figure 3), a Box–Behnken design (Design-Expert 25, Stat-Ease, Minneapolis, USA) with 15 experimental runs was conducted to optimise the ternary film formulation. The experimental design and results are presented in Table 1. The comprehensive K-values ranged from 0.2614 to 0.8116, indicating substantial variation across formulations.

Multiple regression analysis of the experimental data yielded a quadratic polynomial model (Table 2). The model F-value of 164.69 (*p* < 0.0001) indicated high statistical significance, while the lack-of-fit F-value of 14.13 (*p* = 0.0668 > 0.05) confirmed model adequacy. The coefficient of determination (*R*^2^ = 0.9965) indicated excellent agreement between predicted and actual values. The adjusted R^2^ further confirmed the model’s predictive capability.

All linear terms (A, B, C), quadratic terms (A^2^, B^2^, C^2^), and interaction terms (AB, AC, BC) were significant at *p* < 0.01, confirming the importance of both individual factors and their interactions. Based on F-values, the influence of factors on K-value followed the order: B (KGM) > C (LGG) > A (PUL), indicating that KGM concentration was the most critical variable affecting film performance. This observation aligns with findings from other polysaccharide-based film studies, where the reinforcing component often exerts the dominant influence on mechanical properties [18]. The adequacy of the model was further confirmed by the non-significant lack-of-fit (*p* = 0.0668), suggesting that the quadratic model adequately captured the relationship between the independent variables and the response [19].

#### 3.2.2. Analysis of Interactive Effects

**Figure 4:**
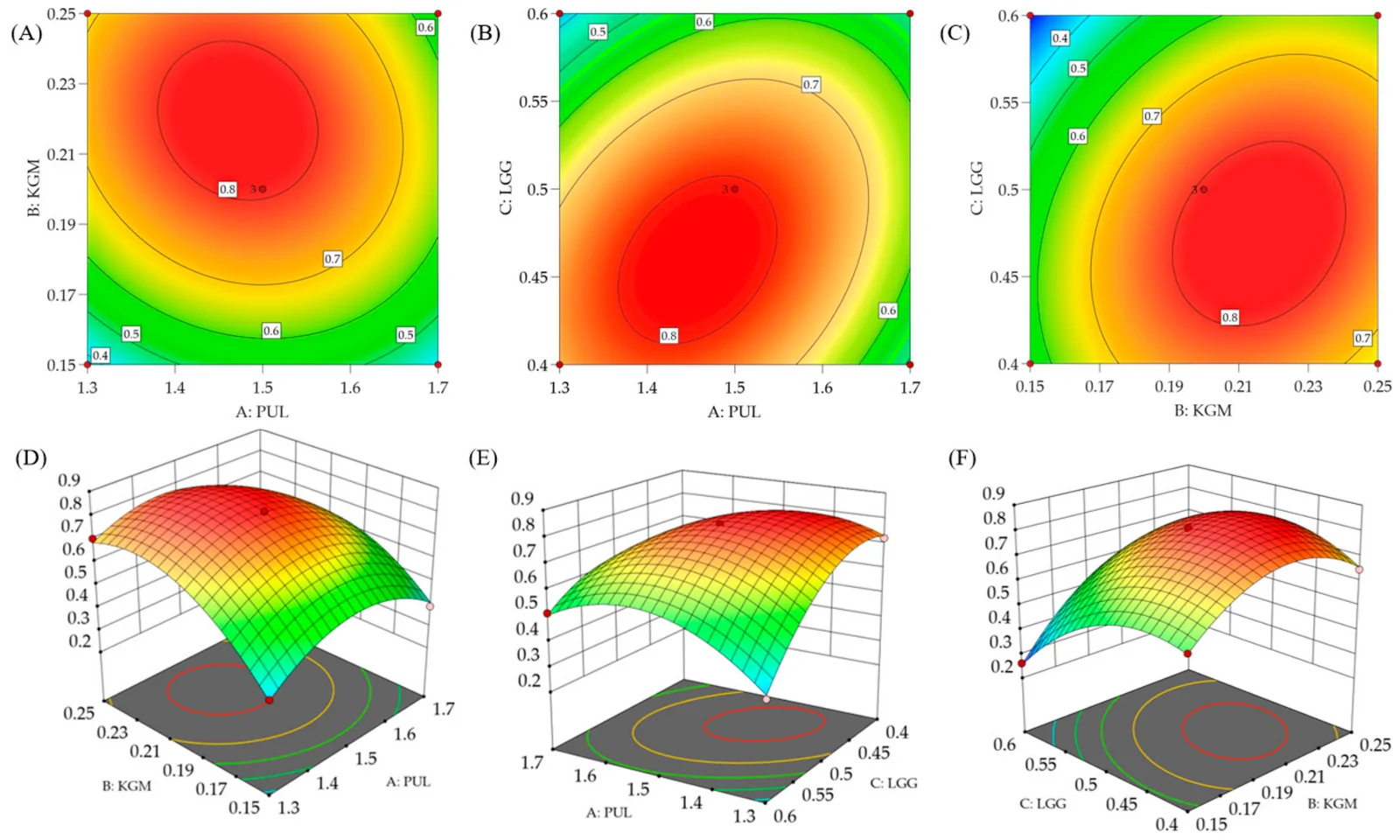
Analysis of the interaction effects on the performance of composite films through 3D graphs and contour plots. Note: (**A**,**D**) represent the interaction effect of the addition amounts of PUL and KGM. (**B**,**E**) represent the interaction effect of the addition amounts of PUL and LGG. (**C**,**F**) represent the interaction effect of the addition amounts of KGM and LGG.

Three-dimensional response surface and contour plots were generated to visualise the interactive effects between variables (Figure 4). The steep curvature and elliptical contour patterns indicated significant interactions among all factor pairs. The PUL–KGM interaction (Figure 4A,D) exhibited a clear maximum at intermediate concentrations of both components, perhaps suggesting synergistic effects in network formation. The K-value increased with increasing PUL and KGM concentrations up to the central region, beyond which excessive polymer loading led to performance decline. This pattern may reflect the balance between hydrogen bonding formation and molecular aggregation [41].

The PUL–LGG interaction (Figure 4B,E) showed a similar convex response surface, with the optimal region centred around 1.5 g/100 mL PUL and 0.5 g/100 mL LGG. The significant AC interaction (*p* = 0.0001) confirms that the combination of PUL and LGG exerts synergistic effects on film performance, possibly through complementary contributions to network integrity and barrier properties [42].

The KGM–LGG interaction (Figure 4C,F) exhibited the steepest response surface among all pairs, consistent with the largest F-value for this interaction. The optimal K-value was achieved at intermediate KGM and LGG concentrations, with excessive levels of either component causing performance deterioration. This finding highlights the importance of balanced polysaccharide ratios for achieving optimal film properties.

#### 3.2.3. Parameter Optimization and Model Verification

Numerical optimisation using Design-Expert software predicted optimal conditions of PUL 1.4263 g/100 mL, KGM 0.2380 g/100 mL and LGG 0.4840 g/100 mL, with a predicted K-value of 0.8141. For practical feasibility, the formulation was adjusted to PUL 1.43 g/100 mL, KGM 0.24 g/100 mL and LGG 0.48 g/100 mL. Validation experiments (*n* = 5) yielded a mean K-value of 0.805 ± 0.013, which agreed closely with the predicted value, confirming model reliability. The optimised film exhibited the following properties: WVP 2.75 ± 0.17 g·μm/(m^2^·h·kPa), light transmittance 89.93% ± 1.26%, TS 3.05 ± 0.14 N/cm^2^, EB 64.57% ± 0.58%.

### 3.3. Mechanical and Barrier Properties of Composite Films

The ternary PUL/KGM/LGG film exhibited substantially superior mechanical performance compared to all binary and single-component counterparts (Table 3), with a TS of 3.05 ± 0.14 N/cm^2^ and an EB of 64.57 ± 0.58%. Relative to pure LGG film (1.33 ± 0.07 N/cm^2^ and 30.14 ± 0.50%, respectively), these values correspond to a 129% improvement in TS and more than a doubling of EB. The EB of the PUL/KGM/LGG film was significantly greater than that of both binary blends (*p* < 0.05), indicating that the ternary combination synergistically enhances extensibility beyond what is achieved by any pairwise mixture. This simultaneous enhancement of both strength and flexibility is of interest, as polysaccharide films typically exhibit an inverse relationship between these two parameters [28]. Film thickness measurements (Table 3) showed that PUL-containing films were slightly but significantly thicker than KGM/LGG and LGG-only films; however, since TS was calculated per unit cross-sectional area and WVP was normalised by thickness, the observed differences in these properties reflect intrinsic material characteristics rather than thickness variations. The balanced improvement can be explained through complementary molecular interactions among the three constituent polysaccharides. Pullulan, with its flexible α-(1→4) and α-(1→6) glycosidic linkages, likely provides chain mobility and extensibility, whereas KGM, possessing a rigid β-(1→4)-linked backbone with branching points, may contribute to network reinforcement through extensive hydrogen bonding [14]. Low-acyl gellan gum, characterised by a double-helix structure that forms strong junctions upon cation-mediated aggregation, could add mechanical strength and thermal stability [12]. In the ternary system, these polymers formed an interpenetrating network wherein pullulan chains acted as flexible connectors between rigid KGM and LGG domains, facilitating stress distribution and energy dissipation during deformation. This hypothesis is supported by FTIR analysis, which revealed the most pronounced peak shifts in the O–H stretching region for the ternary film, suggesting the strongest hydrogen bonding interactions among all formulations.

The ternary film also demonstrated the lowest WVP (Table 3) among all tested formulations, with a value of 2.75 ± 0.17 g·μm/(m^2^·h·kPa). This value was significantly lower than those of PUL/LGG (3.28 ± 0.10), KGM/LGG (3.49 ± 0.09) and pure LGG (3.87 ± 0.11) films (*p* < 0.05). Quantitative comparison with relevant literature further demonstrates the enhanced barrier performance of the ternary system. The WVP of the optimised PUL/KGM/LGG film (2.75 g·μm/(m^2^·h·kPa)) is 28.9% lower than that of pure LGG film (3.87 g·μm/(m^2^·h·kPa)) and 27.3% higher than the value reported by Yan et al. [14] for KGM/PUL binary films (mass ratio 2:1, approximately 2.0 g·μm/(m^2^·h·kPa) when recalculated to comparable units). It is also notably lower than the WVP values (4.1–6.2 g·μm/(m^2^·h·kPa)) reported by Zhou et al. [15] for KGM/PUL films incorporating nanoemulsions. Furthermore, the WVP of the present ternary film compares favourably with values reported for other polysaccharide-based edible films, including pullulan-based films (0.28–0.63 g·μm/(m^2^·h·kPa)) and sodium alginate/pectin blend films (0.84–1.73 g·μm/(m^2^·h·kPa)) [33,36]. The WVP of PUL/LGG and KGM/LGG were not significantly different from each other (*p* > 0.05), but both were significantly lower than that of pure LGG and significantly higher than that of the ternary film. This 28.9% reduction compared to pure LGG is likely attributable to the formation of a denser and more compact microstructure in the three-component system, a finding that correlates well with SEM observations showing a continuous, defect-free structure with minimal microvoids. The incorporation of PUL and KGM may have filled interstitial spaces within the LGG network, creating a more tortuous diffusion path for water molecules and thereby effectively impeding water vapour transmission [42].

Regarding optical properties, all film systems exhibited light transmittance values exceeding 88%, indicating excellent transparency suitable for food packaging applications where visual product inspection is desired. Statistical analysis revealed no significant differences among any of the groups for transmittance (*p* > 0.05), with values ranging from 88.50% to 90.30%. The ternary film showed a slight reduction in transmittance (89.93%) compared to pure LGG (90.30%), which may reflect minor light scattering effects arising from the more complex microstructure; however, this difference is not statistically significant (*p* > 0.05).

### 3.4. Microstructural Characterisation of PUL/KGM/LGG Composite Films

#### 3.4.1. Microscopic Morphology Analysis

**Figure 5:**
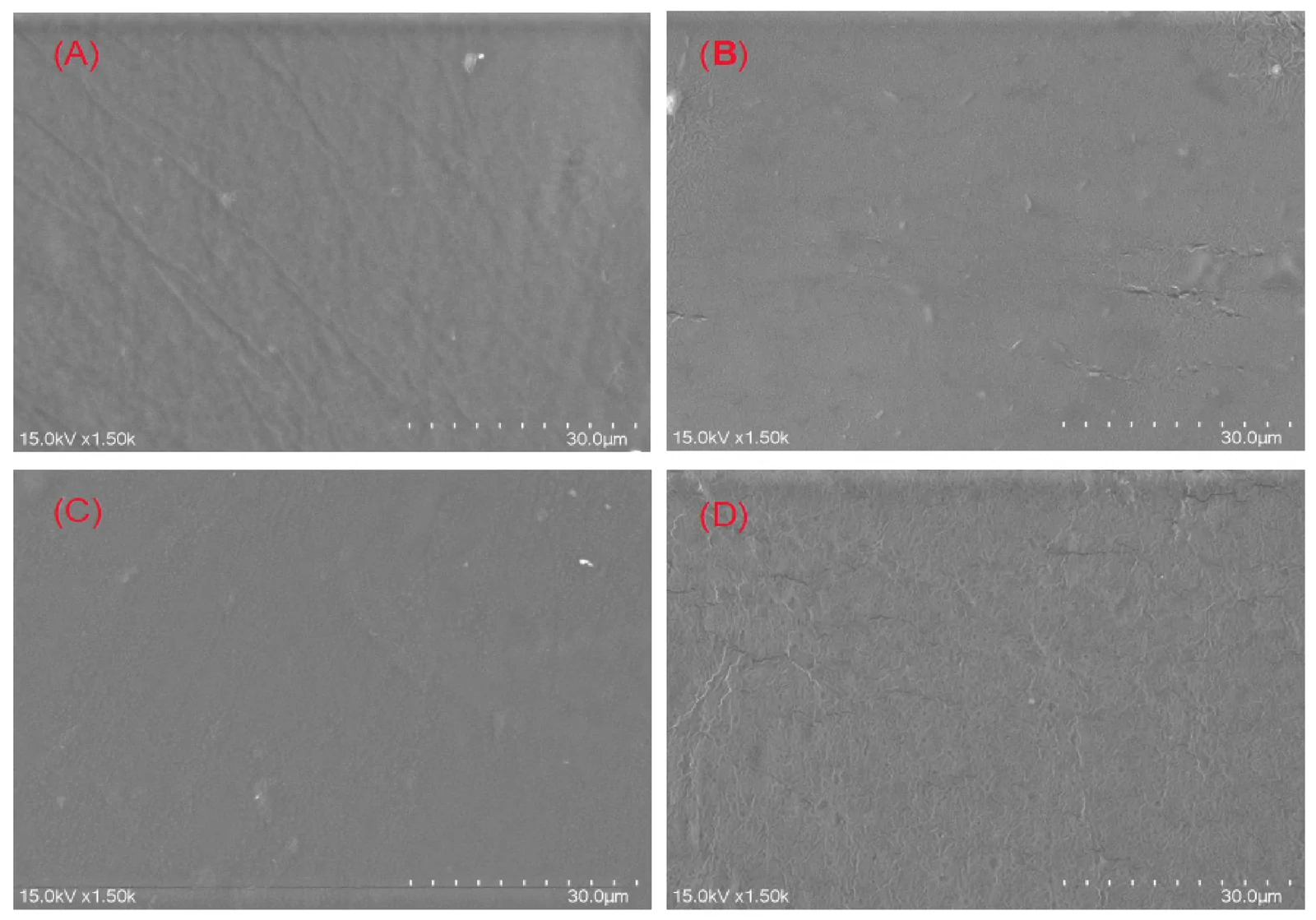
SEM micrographs of composite films. (**A**) Pure LGG film; (**B**) KGM/LGG composite film; (**C**) PUL/LGG composite film; (**D**) PUL/KGM/LGG composite film.

SEM images of pure LGG, KGM/LGG, PUL/LGG and PUL/KGM/LGG films are shown in Figure 5. Pure LGG film exhibited a rough surface with pronounced wrinkles and deep linear cracks, which may reflect the brittle nature and limited molecular interactions of the single-component system. The KGM/LGG binary film showed reduced cracking with finer, more uniformly distributed fissures, which suggests that KGM partially mitigated internal stresses during drying through hydrogen bonding [43]. However, minor micro-cracks persisted, possibly indicating suboptimal compatibility. The PUL/LGG binary film displayed a smooth, dense and homogeneous surface with no visible cracks or defects, suggesting good compatibility between PUL and LGG. This observation is consistent with the ability of PUL to form continuous, well-organised molecular networks with other polysaccharides [44]. The PUL/KGM/LGG ternary film exhibited the most refined microstructure, characterised by a fine, uniform reticular network with shallow, dispersed fissures. The absence of visible pores or fractures indicates the formation of a dense, continuous structure. This enhanced microstructure likely results from synergistic interactions among all three components: PUL may improve system compatibility and film density, KGM could enhance molecular chain entanglement and toughness, and LGG might contribute network rigidity [36]. The complementary roles of the three polysaccharides appear to enable the formation of a structurally superior film suitable for food packaging applications.

#### 3.4.2. FTIR Spectroscopy Analysis

**Figure 6:**
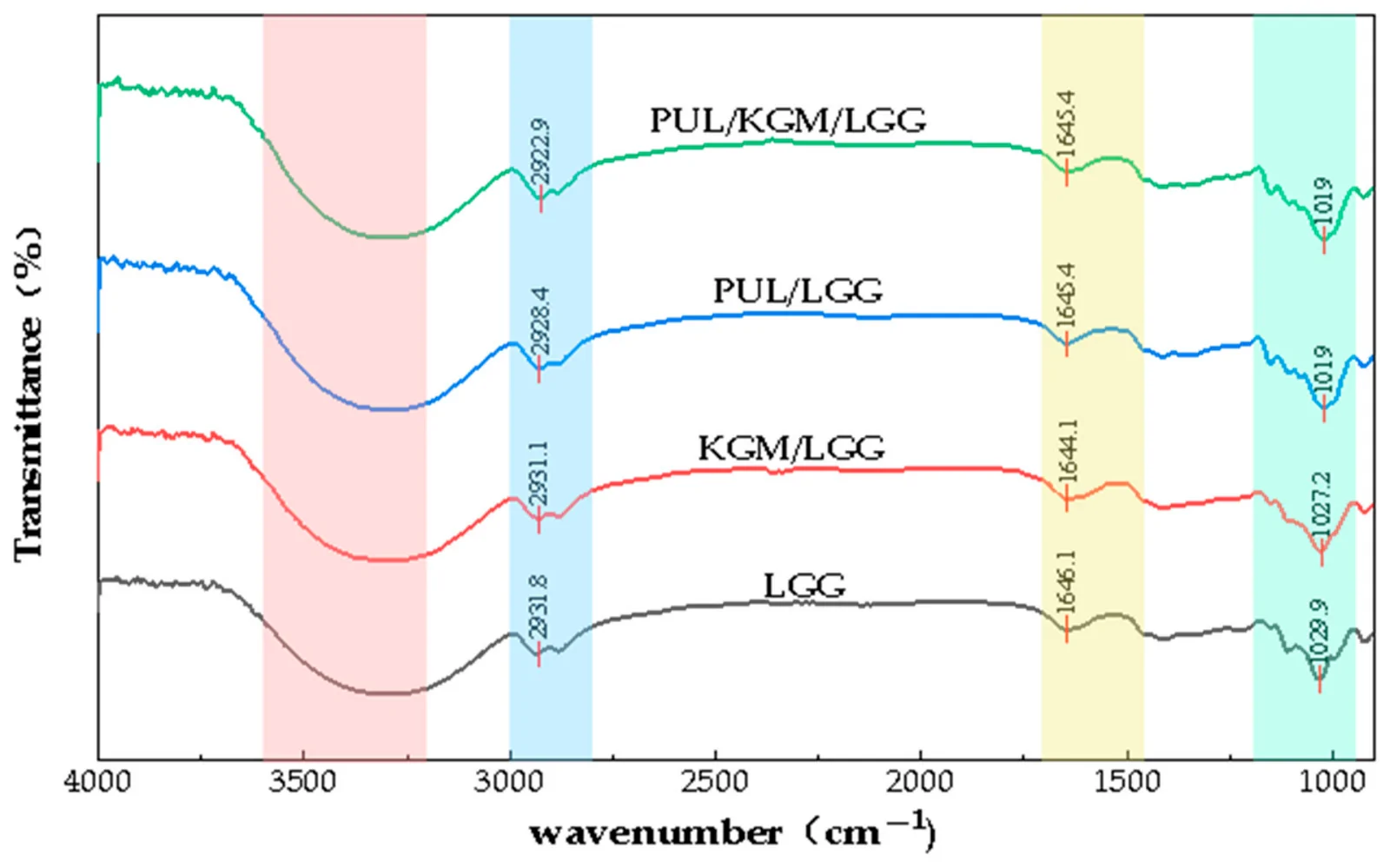
FTIR spectra of composite films.

FTIR spectra of pure LGG, KGM/LGG, PUL/LGG and PUL/KGM/LGG films are presented in Figure 6. All films exhibited characteristic polysaccharide absorption bands. The broad peak at approximately 3400 cm^−1^ corresponds to O–H stretching vibrations, which may be indicative of extensive hydrogen bonding within the film matrix [45]. The band at 3000–2800 cm^−1^ is attributed to C–H stretching vibrations of the polysaccharide alkyl backbone [46]. The peak at approximately 1640 cm^−1^, associated with bound water O–H bending and carboxylate (–COO^−^) stretching of LGG, was clearly observed in all LGG-containing films. The fingerprint region at 1200–950 cm^−1^, corresponding to glycosidic bond and C–O stretching vibrations, confirmed the integrity of the polysaccharide backbone structures [47]. In the PUL/KGM/LGG ternary film, the hydroxyl stretching band exhibited the greatest broadening and shifted to lower wavenumbers compared to binary and single-component films, suggesting enhanced hydrogen bonding interactions among the three polysaccharides [48]. The carboxylate peak at 1640 cm^−1^ remained clearly visible, confirming that LGG’s structural features were preserved in the ternary matrix. These spectral changes may suggest that the three polysaccharides formed a stable, well-integrated network through intermolecular hydrogen bonding, without compromising individual component structures [49].

### 3.5. Functional Properties of PUL/KGM/LGG Composite Films

#### 3.5.1. Antioxidant Activity Analysis of Composite Films

**Figure 7:**
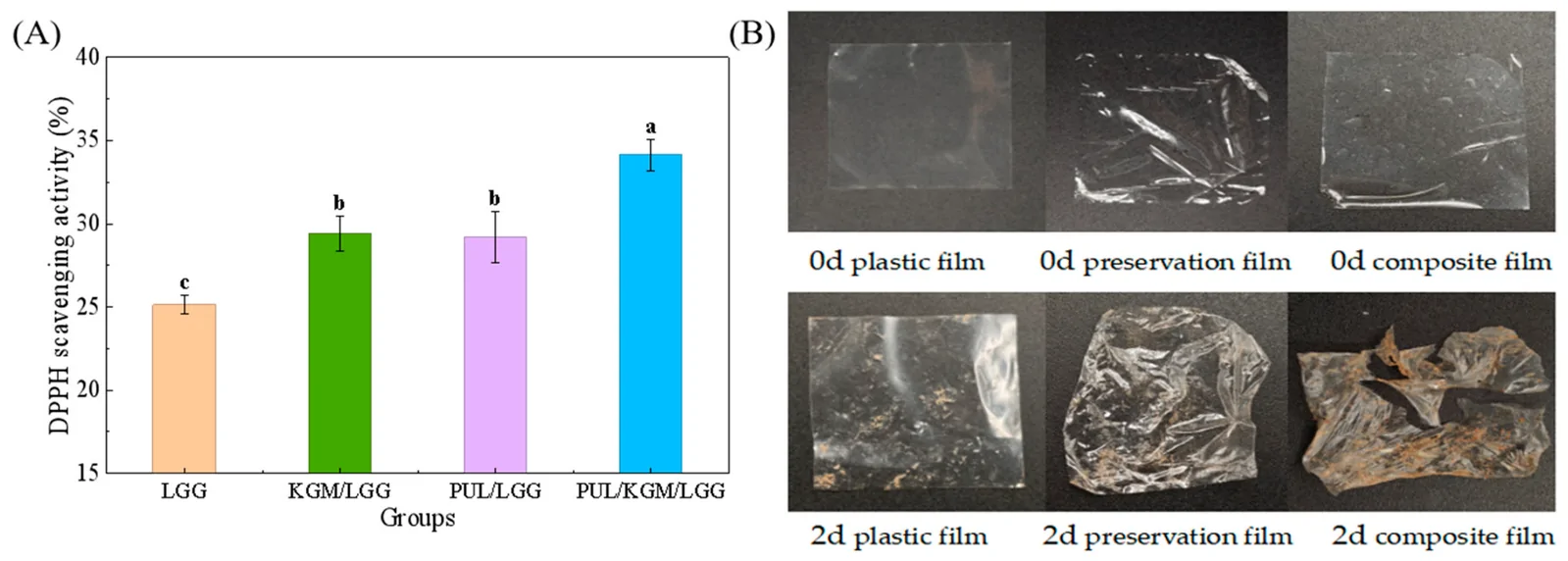
Antioxidant and disintegration properties of composite films. (**A**) represents DPPH scavenging activity of different composite films. (**B**) represents biodisintegration diagrams of different composite films. Different letters indicate significant differences among concentration treatments (*p* < 0.05).

The DPPH radical scavenging activities of the different film-forming systems are presented in Figure 7A. The ternary PUL/KGM/LGG film exhibited the highest antioxidant activity (34.12 ± 0.96%), which was significantly greater than that of all other formulations (*p* < 0.05). The PUL/LGG (29.20 ± 1.56%) and KGM/LGG (29.41 ± 1.06%) binary films showed similar levels of activity, with no statistically significant difference between them (*p* > 0.05), but both were significantly higher than that of pure LGG (25.15 ± 0.56%, *p* < 0.05). These results indicate that the addition of PUL and KGM individually contributes to enhanced radical scavenging, while their ternary combination yields the most pronounced effect.

To clarify the origin of antioxidant activity, we note that the film contains no added exogenous antioxidants (such as essential oils or plant extracts). Therefore, the observed DPPH scavenging activity is an inherent property of the polysaccharide matrix itself, primarily attributed to the hydrogen-donating ability of hydroxyl groups present in PUL, KGM and LGG, which can terminate radical chain reactions [50]. The enhanced activity in the ternary film could arise from the higher density of hydroxyl groups available for radical stabilisation in the more compact ternary network, as well as from synergistic radical-scavenging effects among the three polysaccharides. Compared with pullulan-based coatings alone, which have been reported to extend the shelf life of Chinese plums by regulating ROS scavenging and cell wall metabolism [51], and with gellan/pullulan films incorporated with *Broussonetia papyrifera* extracts, which exhibited 77.45% DPPH scavenging [52], the activity of the present film is moderate, but it is comparable to or higher than that of other polysaccharide-based films containing low levels of natural additives (typically 20–30%) [53]. The comparable performance of PUL/LGG and KGM/LGG binary films suggests that, when combined with LGG, both PUL and KGM contribute equally to the antioxidant capacity. In contrast, the triple combination allows for additional hydrogen-bonding interactions that further enhance electron or hydrogen donation. The moderate antioxidant capacity may nevertheless contribute to preservation efficacy by mitigating oxidative stress in coated fruits. Huang et al. [16] previously reported that KGM/LGG-based coatings delayed senescence-related oxidative damage in blueberries by reducing malondialdehyde accumulation and enhancing antioxidant enzyme activities. Similarly, the antioxidant activity of the PUL/KGM/LGG film could help retard oxidative browning and nutrient disintegration in plums during postharvest storage, complementing its physical barrier and antimicrobial functions to collectively extend shelf life.

#### 3.5.2. Soil DisintegrationProperties of Composite Films

Soil burial tests revealed a disintegration rate of 33.36 ± 0.21% for the PUL/KGM/LGG composite film after 48 h (Figure 7B), substantially higher than that of commercial plastic films (3.98% ± 0.12%). The disintegrated film exhibited visible surface fragmentation and structural collapse, supporting its high susceptibility to soil disintegration [2]. The disintegration process probably proceeds through two overlapping stages: initial water absorption and swelling of the hydrophilic matrix facilitate microbial colonisation, followed by enzymatic hydrolysis of glycosidic bonds in PUL, KGM and LGG, with the resulting oligosaccharides mineralised to CO_2_ and H_2_O [9]. The rapid disintegration observed is consistent with the inherent susceptibility of these polysaccharides to microbial enzymes, including pullulanases, glucomannanases and gellan lyases [6,9]. The ternary network does not appear to hinder soil disintegration; rather, its uniform microstructure may offer an accessible surface for enzymatic attack. This disintegration rate compares favourably with other polysaccharide-based films reported in the literature [54].

This rapid disintegration in soil environments underscores the environmental benefits of the developed film compared to conventional petroleum-based packaging. However, disintegration rates may vary depending on soil type, moisture, temperature and microbial community composition, warranting further investigation under diverse environmental conditions. Overall, the PUL/KGM/LGG film demonstrates strong potential as a sustainable, biodegradable alternative for food packaging applications.

#### 3.5.3. Bacteriostatic Activity of Composite Films

**Figure 8:**
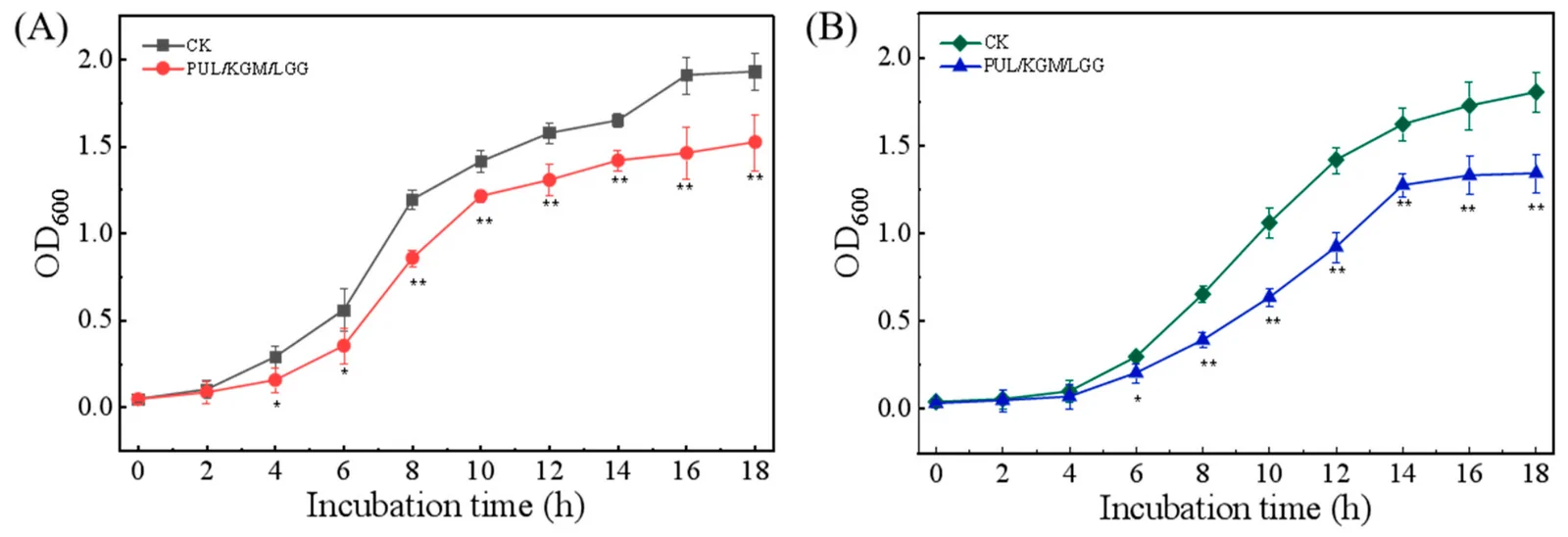
Inhibitory effect of PUL/KGM/LGG composite film on microorganisms. (**A**) *Escherichia coli*. (**B**) *Staphylococcus aureus*. Asterisks indicate statistically significant differences between treatments at the same time point: ‘*’ denotes *p* < 0.05, and ‘**’ denotes *p* < 0.01.

The bacteriostatic activity of the PUL/KGM/LGG film-forming solution against *E. coli* and *S. aureus* was evaluated through growth curve analysis (Figure 8). Both bacterial strains exhibited typical S-shaped growth curves, while the treatment groups showed reduced growth rates during the logarithmic phase compared to controls, indicating that the composite film exerted modest growth-inhibitory effects against both pathogens. The inhibitory effect was more pronounced against *S. aureus* (Gram-positive) than against *E. coli* (Gram-negative), which is consistent with the generally higher susceptibility of Gram-positive bacteria to polysaccharide-based antimicrobial systems as reported by Ahmad et al. [29].

The observed growth inhibition is likely attributable to multiple interrelated mechanisms acting in concert. The high-viscosity polysaccharide network may restrict bacterial motility and reduce the efficiency of nutrient acquisition, while the hydrogen-bonded three-dimensional structure of the film appears capable of adhering to bacterial surfaces, thereby forming a physical barrier that impedes nutrient uptake and metabolite excretion [14,29]. In addition, dissociated ions released from LGG may subtly alter the ionic strength and osmotic pressure of the surrounding microenvironment, an effect that could particularly compromise the cell wall stability of Gram-positive organisms [12]. The outer membrane of *E. coli* is known to confer stronger tolerance to such environmental stresses, which may account, at least in part, for the comparatively weaker inhibitory response observed against this strain. It should be noted that these results are based on optical density measurements and reflect bacteriostatic effects; standard disk-diffusion assays were not performed, and no claim of bactericidal activity is made. Although the antimicrobial effect is moderate, it likely contributes to the overall preservation functionality of the film by delaying the onset of microbial spoilage during fruit storage.

### 3.6. Application of PUL/KGM/LGG Composite Film on Plums

#### 3.6.1. Effect of the Composite Coating on Fruit Weight Loss Rate

**Figure 9:**
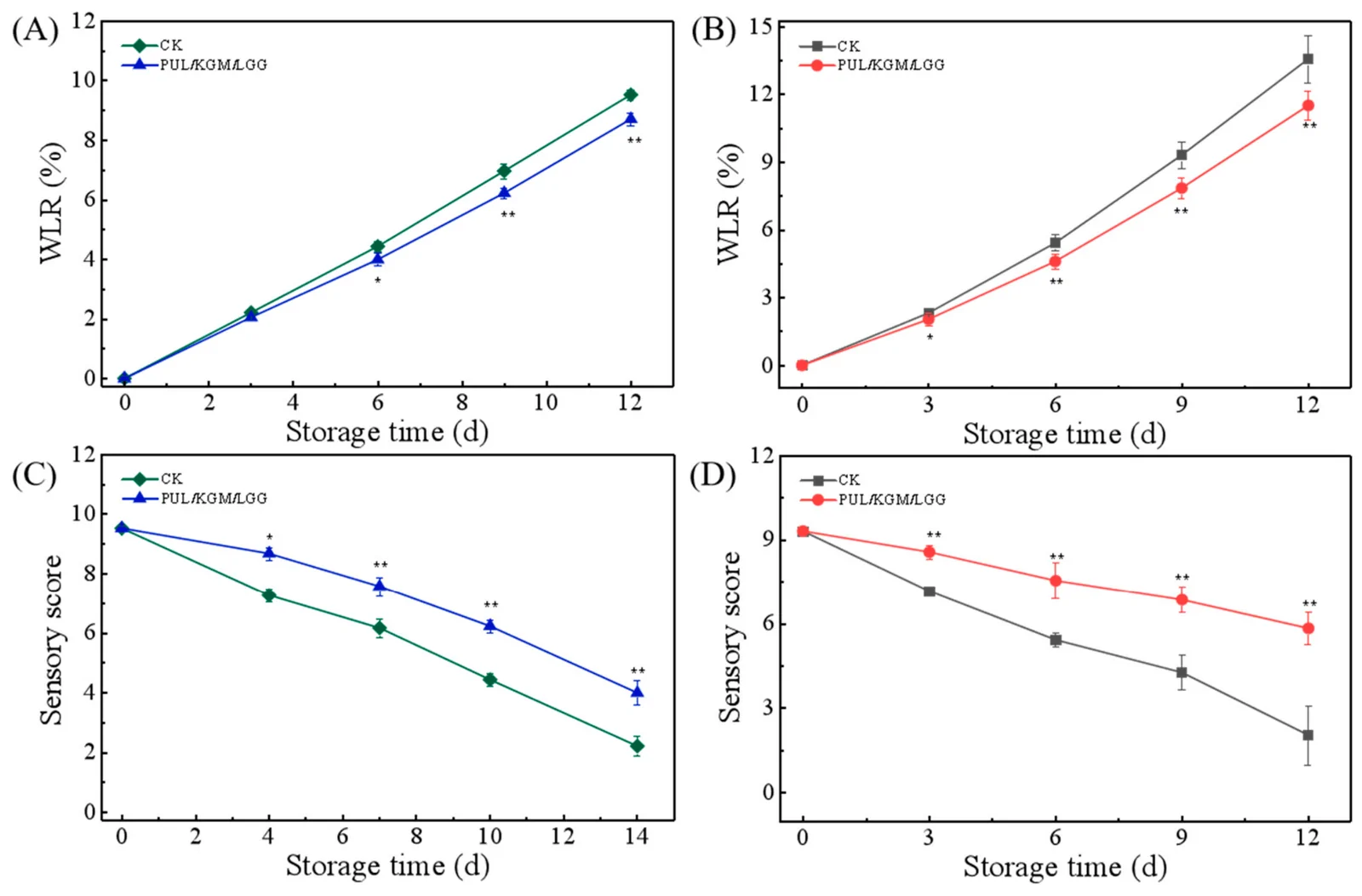
Effect of the PUL/KGM/LGG composite coating on the weight loss rate (WLR) and sensory score of plums. (**A**,**C**) Cultivar Qingcuili; (**B**,**D**) cultivar Yinhongli. Asterisks indicate statistically significant differences between treatments at the same time point: ‘*’ denotes *p* < 0.05, and ‘**’ denotes *p* < 0.01.

Weight loss rate (WLR) during storage reflects moisture loss and respiratory substrate consumption, both of which critically affect fruit quality and shelf life. Figure 9A,B shows the WLR profiles of ‘Yinhongli’ and ‘Qingcuili’ plums during 12 days of ambient storage. For both cultivars, weight loss increased progressively with storage time, but the coated groups consistently exhibited lower weight loss than the control groups. At day 12, the control group of ‘Yinhongli’ showed 13.5% weight loss compared to 11.6% for the coated group; for ‘Qingcuili’ plums, the corresponding values were 9.5% and 8.7%. Statistical analysis revealed significant differences between coated and control groups from day 6 onwards for both cultivars (*p* < 0.05), with the largest absolute differences reaching 1.9 percentage points for ‘Yinhongli’ (day 12) and 0.8 percentage points for ‘Qingcuili’ (day 12). The difference between coated and control groups widened with storage time, possibly indicating sustained barrier effectiveness of the composite film. The reduced weight loss in coated fruits is likely attributed to the formation of a continuous, semi-permeable film on the fruit surface that limits water vapour transmission and respiratory gas exchange. This barrier effect may effectively retard moisture loss and reduce metabolic substrate consumption, thereby preserving fruit fresh weight. The results are consistent with previous reports on polysaccharide-based coatings for plum preservation [22]. Huang et al. [16] similarly demonstrated that KGM/LGG-based coatings effectively maintained fruit firmness and reduced weight loss in blueberries during low-temperature storage by regulating cell wall polysaccharide metabolism.

#### 3.6.2. Effect on Sensory Quality

**Figure 10:**
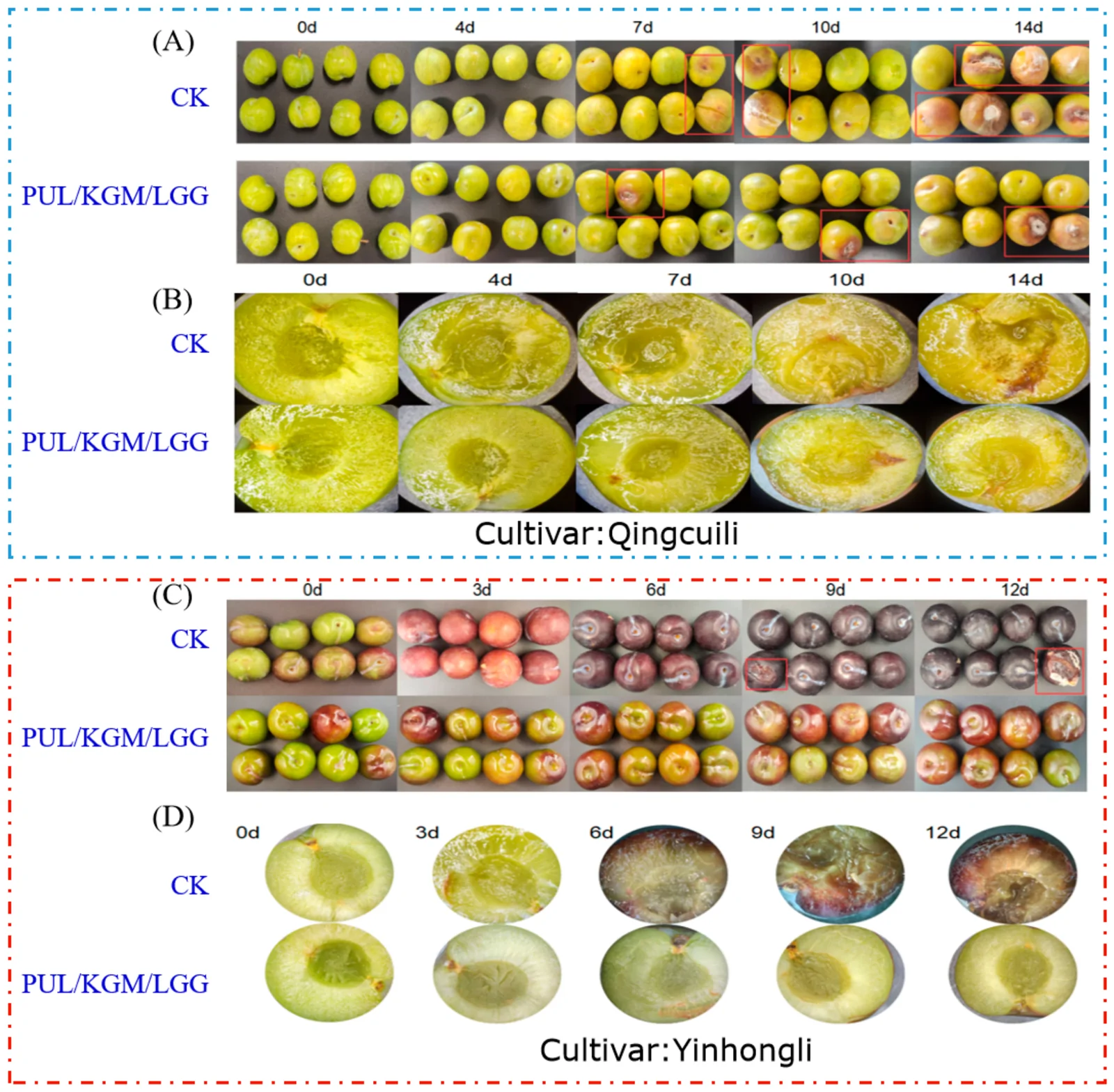
Effects of coating on the appearance quality of plum fruits during storage. Panels (**A**,**B**) represent the ‘Qingcuili’ cultivar, while panels (**C**,**D**) represent the ‘Yinhongli’ cultivar.

Visual assessment of plum sensory quality during storage is presented in Figure 10. For ‘Qingcuili’ plums (Figure 10A), control fruits exhibited progressive loss of gloss, development of brown spots, surface shrivelling and obvious softening by day 12. In contrast, coated fruits maintained a plump appearance, bright colour and firm texture throughout the storage period, with significantly less shrivelling and decay. For ‘Yinhongli’ (Figure 10B), control fruits showed accelerated colour change from green to deep purple-brown, accompanied by pronounced flesh browning. Coated fruits exhibited significantly delayed colour transition and maintained brighter, more appealing colouration. The difference between coated and control groups became statistically significant from day 3 for ‘Yinhongli’ and from day 4 for ‘Qingcuili’ (*p* < 0.05), with the coating showing a more pronounced protective effect on ‘Yinhongli’ plums (Figure 9C,D). At the end of storage, the coated ‘Yinhongli’ and ‘Qingcuili’ fruits achieved total sensory scores of 5.83 ± 0.56 and 3.99 ± 0.41, respectively, which were 2.86-fold and 1.81-fold higher than those of the corresponding control groups. The preservation of sensory quality is possibly attributed to the multiple protective functions of the PUL/KGM/LGG coating. The film may act as a physical barrier against water loss and gas exchange, thereby retarding chlorophyll disintegration, anthocyanin accumulation and enzymatic browning reactions. Additionally, the moderate antioxidant activity of the film could help mitigate oxidative stress and delay senescence. The combined effects of moisture retention, gas barrier and antioxidant protection may enable the composite film to effectively extend the postharvest shelf life of plums. These findings align with the work of Huang et al. [16], who reported that KGM/LGG-based edible coatings suppressed the expression of cell wall-degrading enzymes and maintained the normal morphological structure of fruit cell walls, thereby prolonging shelf life.

## 4. Discussion

To explicitly link film properties to preservation outcomes, the following mechanisms are proposed: (i) the low WVP (2.75 g·μm/(m^2^·h·kPa)) directly reduces moisture loss, as evidenced by the 1.9 percentage point lower weight loss in coated ‘Yinhongli’ plums at day 12; (ii) the high light transmittance (≈90%) allows visual colour assessment but does not directly affect oxidation; rather, the moderate antioxidant activity (34.12% DPPH scavenging) helps neutralise reactive oxygen species, thereby retarding enzymatic browning and pigment disintegration; (iii) the dense, continuous microstructure (SEM) provides an effective gas barrier, reducing oxygen penetration and thus slowing respiration and ethylene production, which in turn delays softening and senescence; and (iv) the adequate mechanical integrity (TS 3.05 N/cm^2^, EB 64.57%) ensures coating adherence and durability during handling, preventing cracks that would compromise barrier function.

The enhanced performance of the ternary film can be explained through complementary molecular interactions among the three constituent polysaccharides. Pullulan, with its flexible α-(1→4) and α-(1→6) glycosidic linkages, likely provides chain mobility and extensibility, whereas KGM, possessing a rigid β-(1→4)-linked backbone with branching points, contributes to network reinforcement through extensive hydrogen bonding. Low-acyl gellan gum, characterised by a double-helix structure that forms strong junctions upon cation-mediated aggregation, adds mechanical strength and thermal stability. In the ternary system, these polymers form an interpenetrating network wherein pullulan chains act as flexible connectors between rigid KGM and LGG domains, facilitating stress distribution and energy dissipation during deformation. This hypothesis is supported by FTIR analysis (Section 3.4.2), which revealed the most pronounced peak shifts in the O–H stretching region for the ternary film, suggesting the strongest hydrogen bonding interactions among all formulations. The presence of glycerol as a plasticiser further modulates the network by intercalating between polysaccharide chains, reducing intermolecular hydrogen bonding and thereby enhancing chain mobility, which explains the improved EB observed in all glycerol-containing films.

Furthermore, the branched structure of KGM contributes to chain entanglements that improve toughness, while the double-helical junctions of LGG, when crosslinked by residual cations, act as physical crosslinking points that enhance the overall network strength. The flexible, random coil conformation of PUL facilitates the even dispersion of the more rigid KGM and LGG domains, thereby preventing macroscopic phase separation. This multi-scale, hierarchical organisation—ranging from hydrogen bonding at the molecular level to microphase separation at the nanoscale—appears to explain which is not typically achieved in single-component polysaccharide films. The findings of Peng et al. [55] on deacetylated konjac glucomannan/high acyl gellan gum water gradient films further support this interpretation, as their work demonstrated that strengthened molecular interactions between KGM and gellan gum resulted in a more structured film matrix with improved barrier properties.

The mechanical properties of the optimised film (TS 3.05 N/cm^2^, EB 64.57%) compare favourably with other polysaccharide-based edible films reported in the literature. For instance, Yan et al. [14] reported TS values of approximately 2.5 N/cm^2^ for KGM/PUL binary films (mass ratio 2:1, total concentration 1% *w*/*v*), while Zhou et al. [15] achieved TS values around 2.8 N/cm^2^ for KGM/PUL films incorporating nanoemulsions. The enhanced performance of the ternary system in the present study—representing a 22% improvement over the KGM/PUL binary system reported by Yan et al. [14]—highlights the benefit of incorporating LGG as a reinforcing component. The WVP of the optimised ternary film (2.75 g·μm/(m^2^·h·kPa)) not only significantly outperformed all control formulations in this study (28.9% reduction vs. pure LGG) but also exceeded the barrier performance of KGM/PUL binary systems reported in the literature by 34.5% [14]. This improvement may be attributed to the ability of LGG to form a more rigid helical structure that interpenetrates with the flexible PUL and KGM chains, thereby creating a more cohesive and load-bearing network. Recent studies on gellan gum and pullulan-based films have also demonstrated that hydrogen bonding interactions between polysaccharides significantly improve mechanical strength and thermal stability. In particular, Yao et al. [17] developed pullulan/gellan gum biofilms loaded with astaxanthin nanoemulsion and reported that the antioxidant and antibacterial effects of the nanoemulsion were maximally preserved during biofilm preparation, indicating the compatibility of pullulan and gellan gum as a film-forming matrix. Ali et al. [51] further showed that the incorporation of Broussonetia papyrifera extracts into gellan gum/pullulan films increased mechanical strength, thermal stability and moisture content, while the extracts were uniformly distributed throughout the matrix via hydrogen bonding. Cheng et al. [56] investigated the effects of two types of gellan gum on the properties of fish gelatin films and found that the addition of gellan gum significantly improved the tensile strength and water vapour barrier properties of the composite films, further supporting the reinforcing role of gellan gum in polysaccharide-based film systems.

The moderate antioxidant activity (34.12% DPPH scavenging) and bacteriostatic activity of the film, while not as potent as films containing exogenous bioactive compounds such as essential oils or plant extracts [38,53], represent inherent functional properties of the polysaccharide matrix. The primary mechanism underlying this antioxidant capacity likely involves the hydrogen-donating ability of the abundant hydroxyl groups in all three polysaccharides, which can terminate radical chain reactions by forming stable phenoxyl radicals [50]. This suggests that the PUL/KGM/LGG system could serve as an effective base for further functionalisation with natural active agents to enhance preservation efficacy. In comparison, Bian et al. [52] reported that gellan gum and pullulan-based films incorporating Broussonetia papyrifera fruit and leaf extracts exhibited significantly enhanced antioxidant properties, with maximum DPPH radical scavenging rates of 77.45% and ABTS scavenging rates of 66.21%. More recently, Yao et al. [17] developed pullulan/gellan gum films loaded with astaxanthin nanoemulsion and reported that the biofilms exhibited high antioxidant activity with an IC_50_ value of 14.87 μg/mL and significant antibacterial effects against both *E. coli* and *S. aureus*, further confirming the compatibility of pullulan and gellan gum as a film-forming matrix. These findings indicate that while the present PUL/KGM/LGG system possesses moderate inherent bioactivity, its capacity as a carrier matrix for exogenous active agents is considerable and warrants further exploration.These findings indicate that while the present PUL/KGM/LGG system possesses moderate inherent bioactivity, its capacity as a carrier matrix for exogenous active agents is considerable and warrants further exploration.

The preservation trials on plums confirmed the practical applicability of the composite film. The reduction in weight loss and maintenance of sensory quality during ambient storage demonstrate that the film effectively addresses the key postharvest challenges of moisture loss and quality deterioration. These findings are consistent with recent studies on polysaccharide-based coatings for plum preservation [24], and extend the application of ternary polysaccharide systems to this commercially important fruit. Huang et al. [16] previously established that KGM/LGG-based coatings effectively delayed postharvest softening in blueberries by regulating cell wall polysaccharide disassembly. Specifically, Yao et al. [17] developed pullulan/gellan gum films loaded with astaxanthin nanoemulsion and demonstrated that the composite films significantly extended the shelf life of strawberries to 18 days at 25 °C, while effectively reducing weight loss, maintaining firmness and delaying the decline of total soluble solids and titratable acidity compared to the control group. Although the present study was conducted under ambient conditions (25 ± 2 °C) rather than refrigerated storage, the observed preservation effects on plums—including reduced weight loss (11.6% vs. 13.5% in control for Yinhongli at day 12) and delayed browning and softening—appear to align with the mechanisms reported by Huang et al. [16]. This suggests that the broader applicability of polysaccharide-based systems extends beyond blueberries to stone fruits such as plums, and that the incorporation of PUL as a third component may further enhance film-forming properties and mechanical integrity. Furthermore, the preservation efficacy observed under ambient conditions is worth noting, as it indicates that the coating can provide meaningful protection even without refrigeration, which may be advantageous for supply chains in developing regions or for short-term retail display. Recent studies have also demonstrated that pullulan-based coatings alone can effectively extend the shelf life of Chinese plums by regulating antioxidant enzyme activities and suppressing cell wall-degrading enzymes, which further supports the potential of PUL-containing ternary systems for plum preservation [51].

A closer examination of the structure–property relationships reveals that the balanced performance of the ternary film does not merely result from additive contributions, but from a synergistic interplay of molecular architectures. The flexible, random coil conformation of PUL is hypothesised to facilitate the even dispersion of the more rigid KGM and LGG domains, thereby preventing macroscopic phase separation. Concurrently, the double-helical junctions of LGG, when crosslinked by residual cations, may act as physical crosslinking points that enhance the overall network strength, while the branched structure of KGM contributes to chain entanglements that improve toughness. This multi-scale, hierarchical organisation—ranging from hydrogen bonding at the molecular level to microphase separation at the nanoscale—could explain the concurrent improvement in both TS and EB, which is rarely achieved in single-component systems. The findings of Peng et al. [55] on deacetylated konjac glucomannan/high acyl gellan gum water gradient films further support this interpretation, as their work demonstrated that strengthened molecular interactions between KGM and gellan gum resulted in a more structured film matrix with improved barrier properties.

Future studies could explore the incorporation of natural active agents (e.g., essential oils, plant extracts, antimicrobial peptides) to enhance the film’s growth-inhibitory and antioxidant functionalities, and evaluate its applicability to a broader range of perishable agricultural products. The inherent compatibility of the PUL/KGM/LGG matrix with bioactive compounds, as suggested by the findings of Yao et al. [17] and Bian et al. [52], provides a strong foundation for such functionalisation strategies.

## 5. Conclusions

A novel ternary edible composite film based on pullulan, konjac glucomannan and low-acyl gellan gum was successfully developed and optimised using single-factor experiments combined with Box–Behnken response surface methodology. The optimised formulation (PUL 1.43 g/100 mL, KGM 0.24 g/100 mL, LGG 0.48 g/100 mL) produced a film with good comprehensive performance, characterised by favourable mechanical strength, low water vapour permeability, high transparency and moderate inherent antioxidant activity. FTIR and SEM analyses confirmed the formation of a dense, continuous microstructure with extensive hydrogen bonding among the three components, validating the synergistic design concept.

The practical application on plums demonstrated that the coating effectively reduces moisture loss, delays browning and softening, and maintains sensory quality under ambient storage, thus addressing key postharvest challenges. The film also exhibits rapid soil disintegration, underscoring its environmental advantage over conventional plastics. While the inherent bioactivity is moderate, the PUL/KGM/LGG matrix shows considerable potential as a carrier for further functionalisation with natural active agents. Overall, this study provides a systematic optimisation framework and mechanistic understanding for developing high-performance ternary polysaccharide films, offering a promising and sustainable alternative for perishable fruit preservation. Future work should explore the incorporation of bioactive compounds and evaluate the film’s performance across a wider range of produce and storage conditions.

## Figures and Tables

**Table 1:** Box-Behnken experimental design and results.

Run	A:PUL	B:KGM	C:LGG	*K*
1	1.5	0.25	0.6	0.6041
2	1.5	0.25	0.4	0.6477
3	1.5	0.15	0.6	0.2614
4	1.3	0.20	0.4	0.7214
5	1.3	0.15	0.5	0.3791
6	1.7	0.20	0.6	0.5101
7	1.3	0.25	0.5	0.7031
8	1.7	0.20	0.4	0.5034
9	1.7	0.25	0.5	0.5428
10	1.5	0.20	0.5	0.8005
11	1.7	0.15	0.5	0.3897
12	1.5	0.20	0.5	0.8116
13	1.3	0.20	0.6	0.3501
14	1.5	0.15	0.4	0.5627
15	1.5	0.20	0.5	0.8087

**Table 2:** Analysis of variance for the response surface model.

Source	Sum of Squares	*df*	Mean Square	*F*	*p*	Significant
Model	0.4361	9	0.0485	164.69	<0.0001	**
A:PUL	0.0054	1	0.0054	18.33	0.0079	**
B:KGM	0.1023	1	0.1023	347.85	<0.0001	**
C:LGG	0.0629	1	0.0629	213.89	<0.0001	**
AB	0.0073	1	0.0073	24.82	0.0042	**
AC	0.0357	1	0.0357	121.42	0.0001	**
BC	0.0166	1	0.0166	56.43	0.0007	**
A^2^	0.0836	1	0.0836	284.25	<0.0001	**
B^2^	0.0862	1	0.0862	292.90	<0.0001	**
C^2^	0.0675	1	0.0675	229.39	<0.0001	**
Residual	0.0015	5	0.0003			
Lack of Fit	0.0014	3	0.0005	14.13	0.0668	
Pure Error	0.0001	2	0.0000			
Cor Total	0.4375	14				

Note: ‘**’ indicates that the difference is very significant, *p* < 0.01.

**Table 3:** Functional properties of different film-forming systems.

Groups	EB %	TS N/cm^2^	WVP g·μm/(m^2^·h·kPa)	Transmittance %	Thickness (mm)
PUL/KGM/LGG	64.57 ± 0.58 ^a^	3.05 ± 0.14 ^a^	2.75 ± 0.17 ^d^	89.93 ± 1.26 ^a^	0.086 ± 0.01 ^a^
PUL/LGG	43.34 ± 0.84 ^b^	1.75 ± 0.11 ^b^	3.28 ± 0.10 ^c^	89.53 ± 1.40 ^a^	0.084 ± 0.01 ^a^
KGM/LGG	38.78 ± 1.46 ^c^	1.71 ± 0.13 ^b^	3.49 ± 0.09 ^bc^	88.50 ± 1.32 ^a^	0.080 ± 0.00 ^b^
LGG	30.14 ± 0.50 ^d^	1.33 ± 0.07 ^c^	3.87 ± 0.11 ^a^	90.30 ± 1.87 ^a^	0.079 ± 0.01 ^b^

Notes: Different lower-case letters in the same column indicate significant differences in the functional indicators among the groups (*p* < 0.05).

## Data Availability

The original contributions presented in the study are included in the article, further inquiries can be directed to the corresponding authors.

## Abbreviations

The following abbreviations are used in this manuscript:

ANOVA	Analysis of Variance
CK	Control Check
DPPH	1,1-Diphenyl-2-picrylhydrazyl
EB	Elongation at Break
*E. coli*	*Escherichia coli*
FTIR	Fourier Transform Infrared Spectroscopy
KGM	Konjac Glucomannan
LB	Luria-Bertani
LGG	Low-Acyl Gellan Gum
PUL	Pullulan
RSM	Response Surface Methodology
*S. aureus*	*Staphylococcus aureus*
SD	Standard Deviation
SEM	Scanning Electron Microscopy
TS	Tensile Strength
WLR	Water Loss Rate
WVP	Water Vapour Permeability

## Appendix A

### Appendix A.1

**Table A1:** Composition of different composite film formulations.

Groups	Polysaccharide Composition (*w*/*v*)
PUL/KGM/LGG	PUL 1.43 g/100 mL, KGM 0.24 g/100 mL, LGG 0.48 g/100 mL
PUL/LGG	PUL 1.43 g/100 mL, LGG 0.48 g/100 mL constitute
KGM/LGG	KGM 0.24 g/100 mL, LGG 0.48 g/100 mL constitute
LGG	LGG 0.48 g/100 mL constitute

### Appendix A.2

**Table A2:** Scoring criteria for sensory quality of plums.

Score Range	Appearance	Odour	Texture	Decay Area
8.1–10.0	Bright, glossy colour; no shrivelling	Intense fresh fruity aroma	Firm and crisp; snaps cleanly	None (0%)
6.1–8.0	Slight discolouration; mild shrivelling	Weak but recognisable fruity notes	Moderately firm; slight indentation recovers	No visible decay
4.1–6.0	Darkened colour; marked shrivelling; sticky surface	Slightly sour or musty off odour	Softened; indentation persists; loss of crispness	5–25%
2.1–4.0	Dull, greyish; severe wrinkles; liquid exudation	Distinct alcoholic or putrid sour odour	Disintegrating; collapses under light pressure	25–50%
0–2.0	Deep dark brown/black; collapse; juice leakage	Intense rotten stench	Mushy/pasty; original texture lost	>50%
